# Cutaneous ultrasound of the nuchal-type fibroma: diagnostic clues and surgery planning

**DOI:** 10.1007/s40477-023-00842-z

**Published:** 2024-01-16

**Authors:** Júlia Verdaguer-Faja, Nuria Rodríguez-Garijo, Carolina Arean-Cuns, Pedro Redondo Bellón, Francisco Javier García-Martínez

**Affiliations:** 1 Department of Dermatology, Hospital Universitari Germans Trias i Pujol, Badalona, Spain; 2 Department of Dermatology, Clínica Universidad de Navarra, Pamplona, Spain; 3 Department of Pathology, Clínica Universidad de Navarra, Madrid, Spain; 4 Department of Dermatology, Clínica Universidad de Navarra, C. del Marquesado de Sta. Marta, 28027 Madrid, Spain

**Keywords:** Nuchal-type fibroma, Cutaneous ultrasound, Ultrasonography, HFUS, Surgery, Mohs micrographic surgery

## Abstract

Nuchal-type fibroma is a rare, benign tumour, arising from the connective tissue and characterized by their usual location in the posterior neck, although extra-nuchal locations may also occur. The excision of nuchal-type fibroma is curative, although it presents as a large poorly circumscribed lesion in the dermal and subcutaneous fat layer, with adipose tissue and muscle fascicles entrapment, what can lead to partial excisions and relapses. Due to its rarity, little is known about the sonographic appearances of nuchal-type fibroma. An early identification and correct extension evaluation is essential to facilitate adequate treatment. Through two clinical cases, we illustrate in this article the utility of cutaneous ultrasound in the early diagnosis of these tumours, highlighting its role in the diagnosis but also in the pre-surgical evaluation improving margins assessment and delimitation.

## Introduction

Nuchal-type fibroma (NTF) is a rare, benign but potentially disfiguring tumour, that usually originates from the posterior cervical region (70%), although one out of three tumours may occur at extra-nuchal sites, especially in face, shoulders, and upper back. It has a strong male predominance (4,5:1) [1–4], and occurs more commonly in the 5th decade of life, although age range is wide [3]. Histologically, it is a poorly circumscribed, subcutaneous to dermal, hypocellular proliferation, with thick and dense collagen bundles and sparse fibroblasts, which are CD34 positive. Characteristically it has ill-defined borders, and might present with entrapment of adipose tissue, skeletal muscle and nerves, making correct surgical and histological margins difficult to assess [1, 3–5]. NTF has been associated with diabetes mellitus, scleredema, and repetitive trauma [3, 5–7]. Besides, Gardner’s syndrome must be ruled out at any diagnosis of NFT, due to its similarities with Gardner fibromas [1, 5, 7].

Due to its rarity, little is known about the sonographic characteristics of NTF. The goal of this article is to describe the sonographic characteristics of two extra-NFT cases of the scalp, and to show its usefulness in diagnosis, pre-surgical evaluation, and margins assessment.

## Clinical cases

### Case 1

**Fig. 1:**
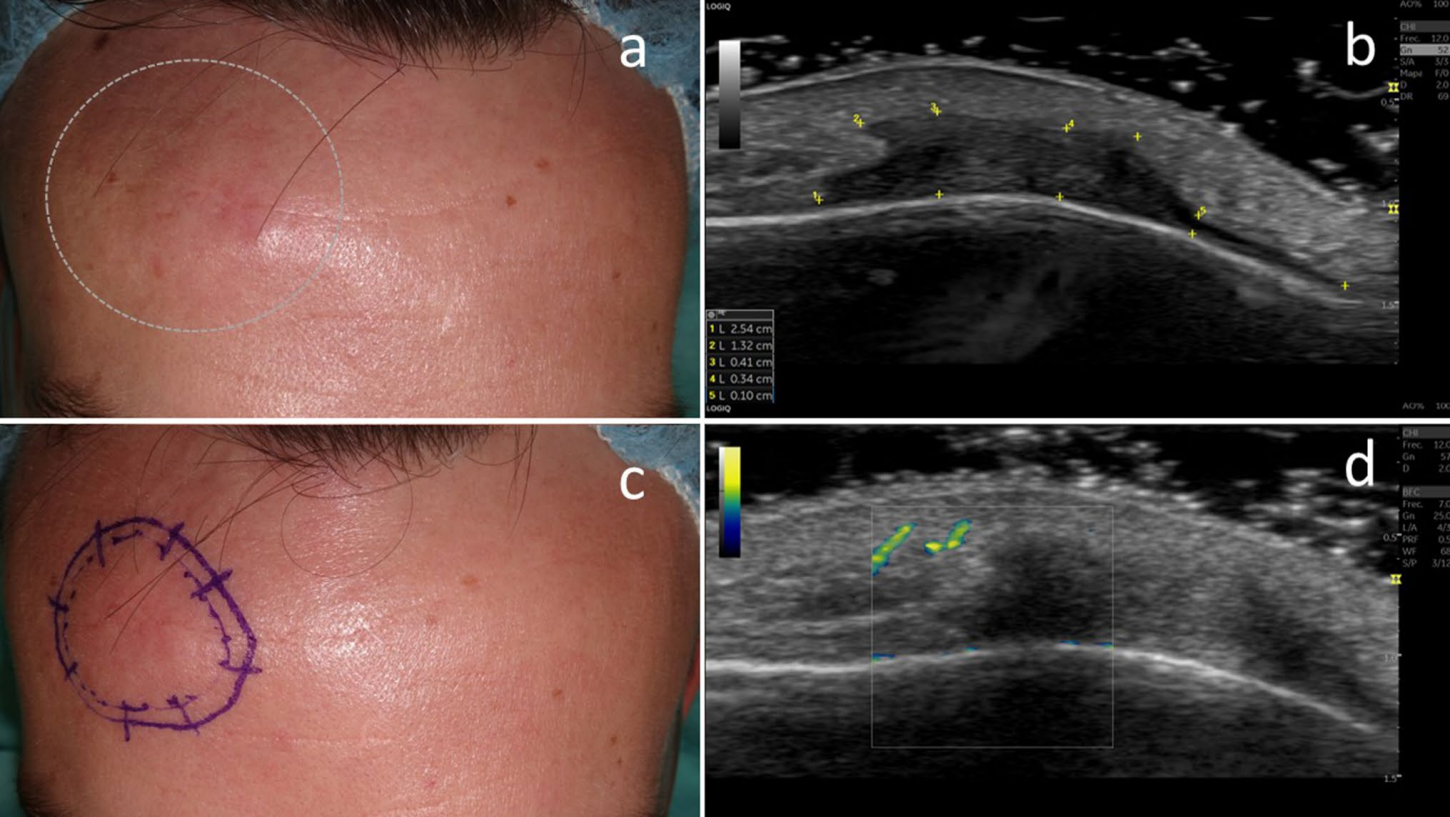
**a** Clinical picture of ill-defined subcutaneous tumour, in the right supraciliary area. **b** Transverse sonographic image showing a poorly defined asymmetric and hypoechoic mass located in dermis-hypodermis, with longest diameters of 2.5 × 0.4 cm. **c** Margins demarcation after ultrasound examination before starting the MMS surgery. Notice that the demarcated area extends beyond what was clinically suspected to the naked eye. **d** Color B-Flow Mode revealed slight increased vascularization exclusively at the periphery of the lesion

**Fig. 2:**
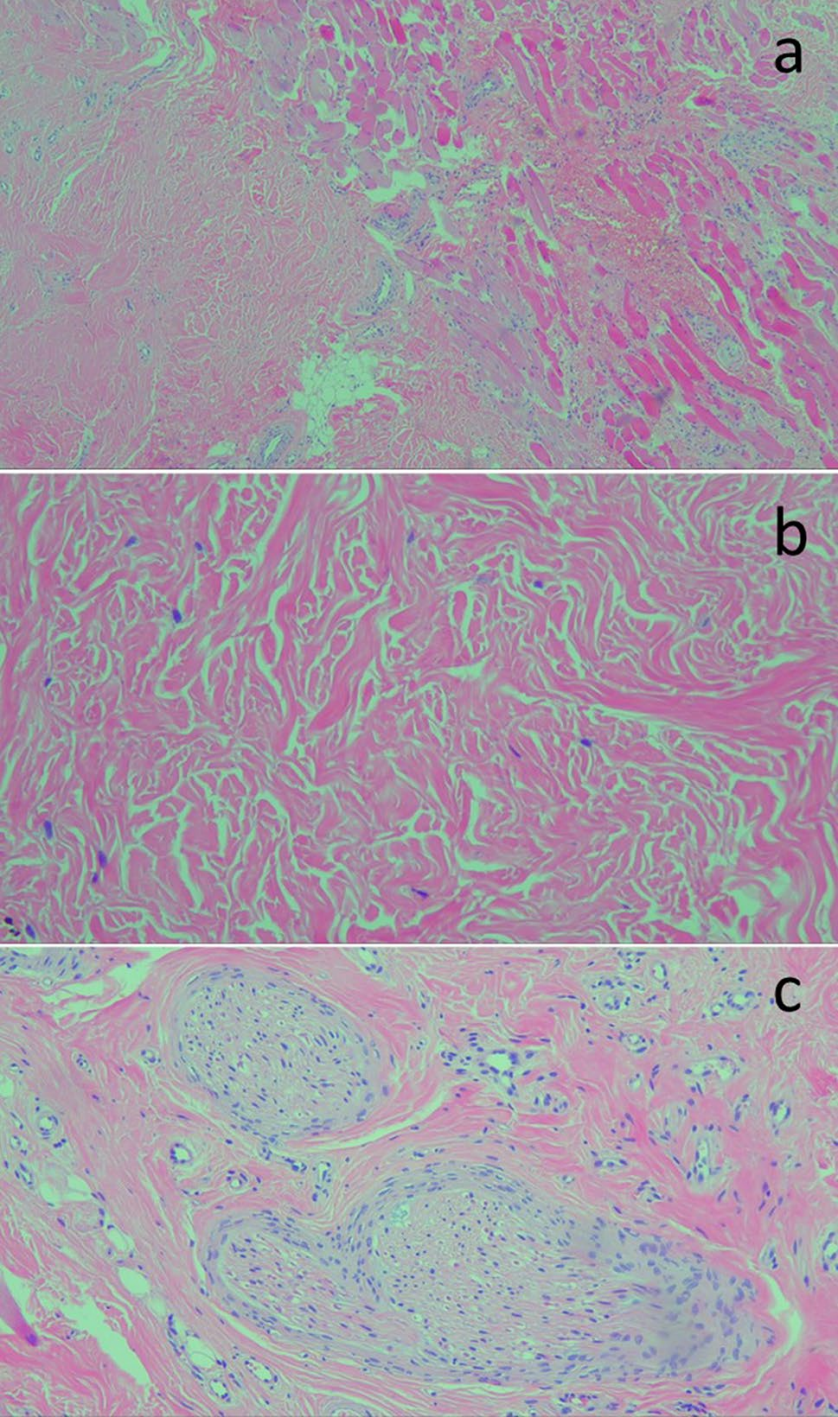
**a** Histological examination showed a poorly demarcated fibroblastic mesenchymal lesion, closely related with the muscular plane (HE, × 40). **b** Thick bundles of collagen with little cellularity corresponding to fibroblasts, with a dense nucleus and without atypia (HE,  × 200). **c** Hypertrophic nerves are detected in the central portion of the lesion (HE, × 100)

A healthy 24-year-old man, presented with a 2-year history of ill-defined subcutaneous tumour of 2 cm, and fixed to deep planes, in the right supraciliary area **(**Fig. 1a**)**. The lesion had a previous excision compatible with NTF. The patient reported early recurrence and rapid growth of the lesion after this intervention. A genetic study was carried out and ruled out associated Gardner syndrome. Longitudinal and cross-sectional sonography scans with L10- to 22-MHz and L8- to 18-MHz probes showed in B-mode a hypoechoic poorly circumscribed lesion in dermis-hypodermis, that expanded below the subcutaneous cellular tissue and frontalis muscle without infiltrating the bone plane (Fig. 1b). Color and Power Doppler mode revealed increased vascularization at the periphery of the lesion, without intralesional flow signal (Fig. 1d, e). Due to these findings, and under suspicion of NFT relapse, surgical excision by Mohs micrographic surgery (MMS) with delayed histologic evaluation (slow MMS) was planned. Ultrasound examination was also used intra-surgery for better margins assessment (Fig. 1c). Histological examination revealed a poorly demarcated lesion composed of thick collagen bundles and entrapped hypertrophic nerves and adipose tissue with variable-sized adipocytes. The sclerosing lesion was paucicellular, and contained CD34-positive, β-catenin-negative fibroblasts infiltrating subcutis (Fig. 2). It stood out the frontal muscle and depressor supercilia involvement, what lead to a secondary paresis at the end of the surgical procedure. After 3 slow MMS stages the complete excision of the tumour was achieved. At 3-years follow-up no signs of recurrence were found.

### Case 2

**Fig. 3:**
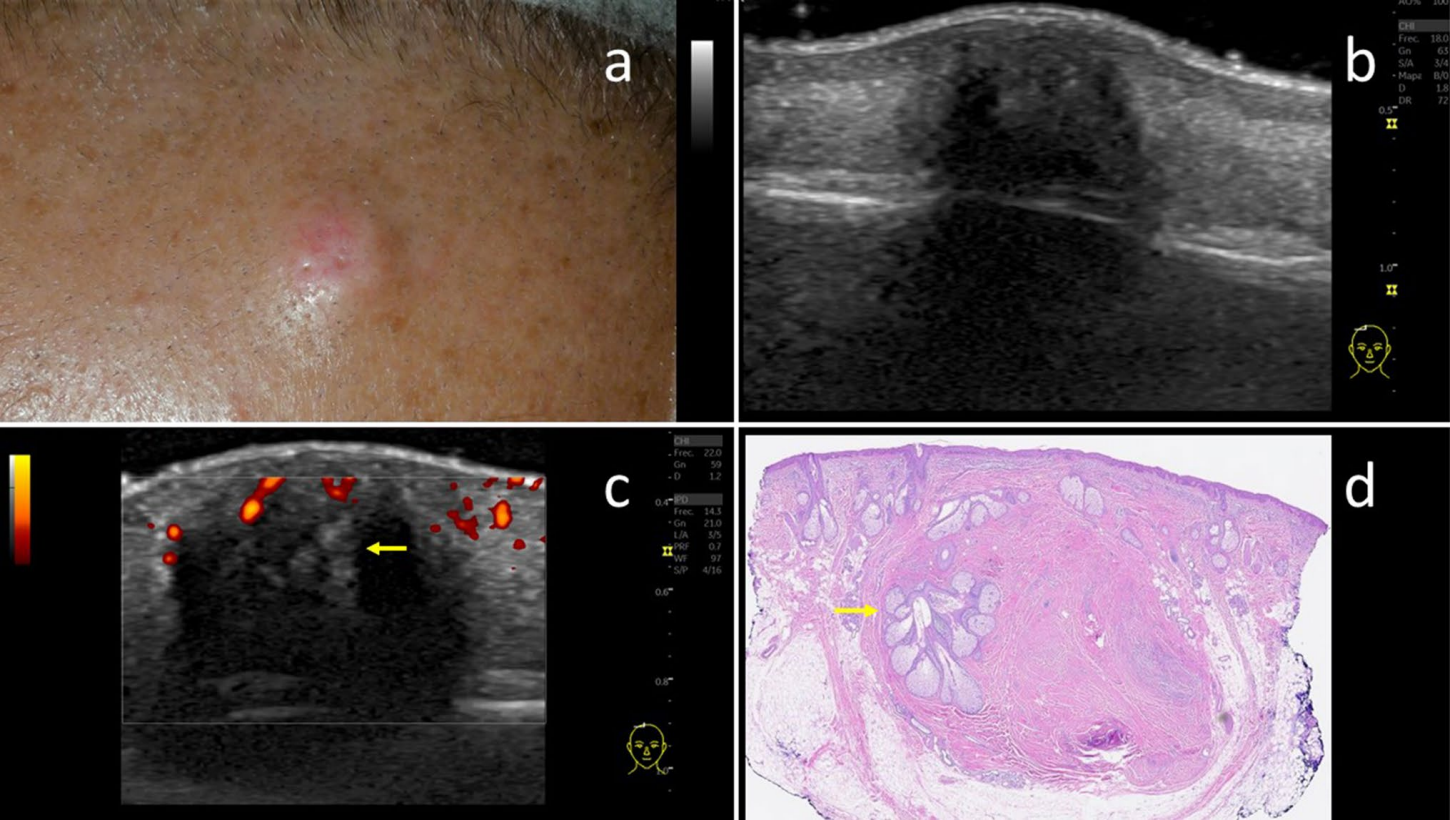
**a** Clinical picture of a round, reddish tumour in the vertex, with maximum diameter of 8 mm, firm and mildly tender on touch, mobile. **b** Ultrasound scan showing an ill-defined hypoechoic nodule ubicated in dermis and hypodermis. **c** Power Doppler mode revealed increased vascularity at the periphery of the lesion, without intralesional vascularization. Notice the hyperechoic image inside, in the upper part, which corresponds to the entrapment of the sebaceous gland also seen in the histological image (yellow arrow). **d** Low magnification microscopic findings (HE). Histological examination shows the presence of a roundish hypocellular proliferation in dermis and hypodermis, with dense, haphazardly arranged, collagen bundles mixed with adipocytes, nerve fascicles and a big sebaceous gland in the left upper side (yellow arrow), correlating with the sonography findings previously described

A 70-year-old man consulted for a 1-year-old lesion in the scalp. Physical examination revealed a round, reddish tumour of 8 mm on vertex, indurated and mildly tender on touch (Fig. 3a). Longitudinal and cross-sectional sonography scans with L10- to 22-MHz and L8- to 18-MHz probes showed in B-mode a hypoechoic ill-defined lesion limited to the dermis-hypodermis (Fig. 3b), and in Color and Power Doppler mode an increased vascularization at the periphery of the lesion (Fig. 3c). A surgical excision was finally performed, with direct closure. Histological examination showed the presence of a roundish hypocellular proliferation in dermis and hypodermis, with dense, haphazardly arranged, collagen bundles mixed with mature adipocytes, sebaceous glands and nerve fascicles; and few spindle cells without atypia within the collagen bundles. Immunohistochemistry demonstrated that the spindle cells were stained positive for CD34. The histological findings were consistent with the diagnosis of NTF, and the resection margins were free (Fig. 3d). 8-month follow-up revealed no signs of relapses.

## Discussion

NTF is a rare, benign tumour, commonly presented as an ill-defined, firm and sometimes disfiguring, subcutaneous nodule appearing in the head and neck region of middle-aged male [1–3]. Surgical excision is the treatment of choice [5]. Histologically, it is an unencapsulated fibrocollagenous tumour characterized by poorly circumscribed borders, and areas with entrapped adipocytes and muscle fascicles, but also nerve fibers often with peripheral traumatic neuroma-like areas [1–3, 5]. These characteristics make correct surgical margins difficult to assess, and recurrences may occur after treatment [3, 5, 7, 8]. In this regard, MMS may be of interest in NTF management, allowing to control margins and avoiding large excisions [7].

NTF is frequently misdiagnosed and under‑reported due to its histopathological similarities with other benign fibrous tumours, including desmoid-type fibromatosis and collagenous fibroma, fibrolipoma, elastofibroma, and dermatofibroma [6, 7]. Although the histological characteristics of NTF could resemble to other fibrocollagenous tumours, knowing its sonographic appearance may help in its diagnosis. For instance, dermatofibroma (the nodular type) shows on ultrasound as a focal dermal thickening and hypoechogenicity, sometimes with a fusiform shape, more deeply in the central part, that may distort the regional hair follicles; while fibrolipomas tend to appear as well-defined oval-shaped hypoechoic hypodermal structures with hyperechoic linear fibrous septa inside, following the axis of the skin layers, and without intralesional vascularity on Color Doppler.

Only a few reports focus on the findings of imaging studies in NTF [5]. MRI has been described in several articles as an efficient modality for differential diagnosis and extension assessment of NTF, due to its superior soft tissue resolution and multi‑planar capabilities. Currently, it is the imaging study of choice. It usually shows an ill-defined mass, with low signal intensity in T1- and T2-weighted images [5, 6]. CT has also been used occasionally, but with less relevance [5, 8].

Nevertheless, we consider that high frequency ultrasonography (HFUS), which has recently been increasingly used and proved it’s utility in dermatology [9–11], could also be useful in NTF’s management. The high resolution of superficial structures available with HFUS can allow the in vivo approximation to a histologic evaluation of the cutaneous structure and its margins, helping in the diagnosis but also in the pre-surgical evaluation, by tumour delimitation and election of the best surgical approach [12].

In the literature review only one article of Møller et al. reported the use of ultrasound examination during NTF diagnosis, describing it as a hypoechoic mass [13]. A second article described the sonography findings of a Gardner fibroma associated to a Gardner’s syndrome, showing some differences: an heteroechoic subdermal mass with traversing internal vessels [14]. Both articles reported the ultrasonography without showing the sonographic images.

To the best of our knowledge, this is the first article describing the sonographic findings of NTF and presenting it with sonographic images. The ultrasound findings in our two patients were comparable between them, and similar to that described by Møller et al. We observed a hypoechoic ill-defined lesion in dermis-hypodermis in B-mode, and increased vascularization at the periphery of the lesions in Color and Power Doppler.

Surgical excision is the mainstay of treatment for NTF [3, 5, 8]. Although NTF are classified as benign tumours, their early diagnosis and complete surgical removal with adequate surgical margins are important to avoid relapses and further locally aggressive surgeries [5]. Skin ultrasound offers a non-invasive and low-risk evaluation, making it a preferred tool for both dermatologists and patients. Furthermore, its availability in most clinical settings makes it accessible and effective in detecting subcutaneous lesions such as NTF. In our experience, the HFUS was useful to suspect the relapse of one of the cases, but above all to assess the tumours size, shape, extension, affected layers and margins delimitation. Besides, we want to highlight its role in MMS surgery planning, which permitted to better predict the extension of the tumour (especially in depth and the muscle involvement), and its correlation with histologic findings.

## Conclusion

In this article we present two cases of NTF and its HFUS findings. Cutaneous ultrasound proved to be useful in the diagnosis but also in the pre-surgical evaluation and margins assessment. Given the growth nature of this tumour, HFUS can be a clue tool to better define the extent of the tumour in the pre-surgical evaluation and guide the MMS surgery.
